# Quick selenium accumulation in the selenium-rich rice and its physiological responses in changing selenium environments

**DOI:** 10.1186/s12870-019-2163-6

**Published:** 2019-12-17

**Authors:** Yuanke Liang, Yang Su, Ling Li, Xin Huang, Faiz Hussain Panhwar, Tengda Zheng, Zhichen Tang, Hla Hla Ei, Muhammad Umer Farooq, Rui Zeng, Yujie Zhang, Xiaoying Ye, Xiaomei Jia, Lanlan Zheng, Jianqing Zhu

**Affiliations:** 1 Crop Genetics and Breeding, Rice Research Institute, Sichuan Agricultural University, Chengdu, 611130 Sichuan China; 2 Laboratory of Medicinal Plant, Institute of Basic Medical Sciences, School of Basic Medicine, Biomedical Research Institute, Hubei Key Laboratory of Wudang Local Chinese Medicine Research, Hubei University of Medicine, Shiyan, 442000 Hubei China; 3 Hubei Key Laboratory of Embryonic Stem Cell Research, Taihe hospital, Hubei University of Medicine, Shiyan, 442000 Hubei China; 4 Dujiangyan Agricultural and Rural Bureau, Dujiangyan, 611830 Sichuan China

**Keywords:** Sodium selenate, Biochemical activity, Physiology, Selenium-rich rice

## Abstract

**Background:**

The element selenium (Se) deficiency is thought to be a global human health problem, which could disperse by daily-supplement from Se-rich food. Increasing the accumulation of Se in rice grain is an approach matched to these nutrient demands. Nonetheless, Se is shown to be essential but also toxic to plants, with a narrow margin between deficiency and toxicity. Notably, the regulatory mechanism balancing the accumulation and tolerance of Se in Se-rich rice plants remains unknown.

**Results:**

In this study, we investigated the phenotypical, physiological, and biochemical alterations of Se-rich rice in the exposure to a variety of Se applications. Results showed that the Se-rich rice was able to accumulate more abundance of Se from the root under a low Se environment comparing to the Se-free rice. Besides, excessive Se led to phytotoxic effects on Se-rich rice plants by inducing chlorosis and dwarfness, decreasing the contents of antioxidant, and exacerbating oxidative stresses. Furthermore, both phosphate transporter *OsPT2* and sulfate transporters *OsSultr1;2* may contribute to the uptake of selenate in rice.

**Conclusions:**

Se-rich red rice is more sensitive to exogenous application of Se, while and the most effective application of Se in roots of Se-rich rice was reached in 20 μM. Our findings present a direct way to evaluate the toxic effects of Se-rich rice in the Se contaminated field. Conclusively, some long-term field trial strategies are suggested to be included in the evaluation of risks and benefits within various field managements.

## Background

Selenium (Se) is an indispensable micronutrient for the health of humans and animals. Studies have shown that Se supplementation enhances the ability to scavenge free radicals, coordinating immune responses and delaying the aging of the immune system [1–3]. Seprevents the cellular senescence process and death through interfering the accumulation of peroxides and scavenging free radicals, thereby reducing or delaying the production of lipofuscin [4]. The uptake of Se is closely related to human Se nutritional status, which appearances that either lack or excess uptake of Se negative impacts human’s health [5, 6]. While the demanded amount of Se to human of Se is extremely low, the abundance of Se is relative rare in the Earth’s crust. Nevertheless, Se deficiency is thought to be a global human health problem, demanding a urgently address [7].

Plant scientists believe that Se is a beneficial element, since it is involved in regulating plant growth and development, ranging from regulating plant photosynthesis and respiration, reducing free radicals damages, enhancing plant stress resistance, to alleviating the heavy metals-induced toxicity [1, 6]. At the same time, it can increase the contents of chlorophyll and carotenoid leaves, reducing damages caused by ultraviolet-induced oxidative stresses [8–10]. It has been demonstrated that Se exhibits either beneficial or toxic effects on plant growth and development in a low or high concentration, respectively. Although the contents of Se vary by species and cultural regions, monitoring and optimizing its quality and concentrations is a promising way to avoid undesirable Se deficiency and toxicity [6, 11]. However, the role of Se in plant physiology has not yet been elucidated [12].

Rice (*Oryza sativa* L.), one of the most important crops, is the main food source to over half of the world’s population and contributes 55–80% to the total calorie in a daily diet [13, 14]. It is meaningful to study the effects of sodium selenate on the growth and physiology in rice. At present, the research on selenization of rice branches mainly into two subjects: one is to improve the content of Se in rice by exogenous application of sodium selenite from the perspective of the betterment of the physiological cultivation of rice; the other one is to generate Se-rich progeny by genetic combination of a variety of Se enriched parents from perspective of traditional breeding [1, 12, 15, 16]. It has shown that the discrimination of genotypes determines the different appearances of the uptake and enrichment of Se in rice [17]. Molecular evidence has suggested that the silicon (Si) transporter protein Lsi1 (OsNIP2; 1) is permeable to selenite in rice [18].The Pi transporters, in particular, OsPT2, catalyzes the uptake of selenite, indicating a similar uptake mechanism shares between selenite and phosphate (Pi) [19].The sulfate transporter SULTR1;2 plays a central and specific role in regulating selenate sensitivity in both *Arabidopsis* and *Stanleyapinnata* [12, 20, 21]. Also, cadmium (Cd) has antagonistic effects to Se [14]. To sum up, it appears that the uptake of Se in plants is mediated by various transporters of ion elements. However, the underlying mechanisms of Se enrichment in the grains of soil-grown Se-rich rice are yet to be revealed.

In this study, the Se-rich red-colored grain hybrid rice Z2057A/CR727 and the sibling Se-free rice CR727 were included to explore the physiological effects of sodium selenate, by evaluating their physiological growth responses to the application of a range of concentrations of Se. The behavior of Se transport was observed in both rice variants, indicating that the Se-rich rice is capable to facilitate the uptake and transportation of Se from roots to leaves. The main rice physiological characters, including the contents of anthocyanin and Se in different parts and biochemical activities, has been determined in 2 d- and 14 d- old plants, providing a better understanding of plant responses and behaviors related to Se status. Regarding to a better physiological plant growth in the existence of Se, an optimized level of sodium selenate should be assessed and applied in the field crop applications.

## Results

### The Se-rich rich is more sensitive to the application of Se than the Se-free rice variety

**Fig. 1:**
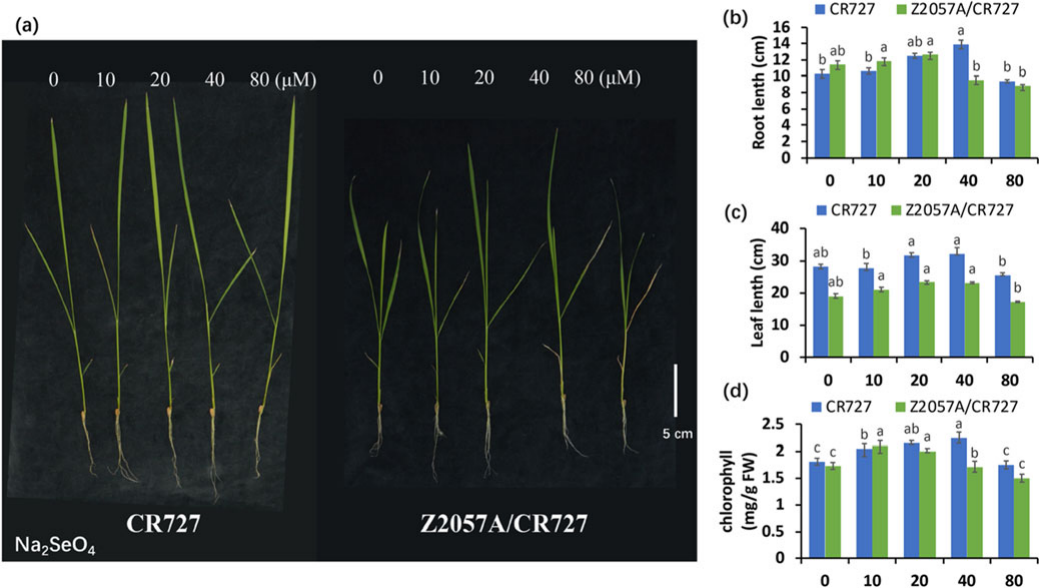
The phenotype observation of seedling (**a**), Root length (**b**), leaf length (**c**), chlorophyll (**d**), phenotype in the presence of exogenous Se. *Bar* = 5 cm, data presents as mean ± SE (*n* = 10, a, b; *n* = 3, c). Different letters indicate significant differences (*p* < 0.05)

To reveal the physiological responses to Se, the seedlings of the Se-rich red-grain rice Z2057A/CR727 and the control Se-free rice CR727 were subjected to a range of exogenous Se treatments. The root growth in both rice variants was promoted in the presence of Se in a dose-dependent manner and impaired in the higher concentration of Se (80 μM) (Fig. 1a and b). With the increasing of Se, the Se-induced increase and inhibition of root elongations occurred earlier in Z2057A/CR727 than in CR727, at 10 μMv*.s.* 20 μM and 40 μM*v.s.* 80 μM, respectively, indicating that the Se-rich rice is more sensitive to the application of Se than its Se-free counterpart. Similar response patterns were demonstrated in the observation of the leaf length, even though the overall leaf lengthofthe Se-rich rice are shorter than that of the Se-free rice in every conditions (Fig. 1a and c), demonstrating that there is a suitable range of exogenous Se to promote growth in a particular rice variety.

We next investigated whether the involvement of Se affects the content of chlorophyll. The results suggested that Se increase the accumulation of chlorophyll in a dose-dependent manner, which is in line with the phenotypes observed in the growth of roots and leaves (Fig. 1d).

To understand how the above physiological alterations occurred in the present of Se, the Se distributions in different organs after a 14 d exposure to exogenous Se were analyzed. With the increasing of Se, the internalization of Se accumulated accordingly in roots, stems, leaf tips and main leaves (Table 1). It was observed that the Se contents increased significantly with the increasing concentrations of sodium selenate in different plant parts. A similar ascending trend for Se accumulation was observed for Se-free rice and Se-rich rice for root and stem parts, while the trend for leaf tips and all leaf produce was different for material under investigation. Under the treatments of 20 μM and 40 μM Se, the accumulation of Se was maximum in the stem, leaf tips, and all leaf produce, while a high concentration of 80 μM decreased the Se accumulation. The result indicated that the Se-rich rice was more sensitive to Se uptake than the Se-free rice.

**Table 1:** The contents of Se in roots, stems, main leaves, and leaf tips in Se-rich rice and Se-free riceafter treatment for 14 d

Selenium content (mg/kg)	Root		Stem		Leaf		Leaf tip	
	CR727	Z2057A/ CR727	CR727	Z2057A/ CR727	CR727	Z2057A/ CR727	CR727	Z2057A/ CR727
0	0.024^e^	0.068^e^	0.032^e^	0.078^d^	0.036^d^	0.084^e^	0.027^e^	0.077^e^
10 μM	1.243^d^	1.528^d^	5.243^d^	8.528^c^	4.327^c^	10.498^d^	3.253^d^	7.367^d^
20 μM	2.984^c^	3.682^c^	8.984^c^	12.283^b^	7.566^b^	20.363^a^	4.358^c^	12.753^a^
40 μM	5.547^b^	5.893^b^	10.546^b^	13.896^a^	11.383^a^	18.652^b^	6.355^a^	10.692^b^
80 μM	6.582^a^	7.491^a^	11.583^a^	12.493^b^	11.159^a^	14.351^c^	5.850^b^	9.494^c^

Lowercase letters (a, b, c, d and e) on the right of the data indicate the statistical significance between different groups according to Duncan’stest (*p* < 0.05)

### The effects of exogenous Se on the aboveground architectures of rice seedlings

**Fig. 2:**
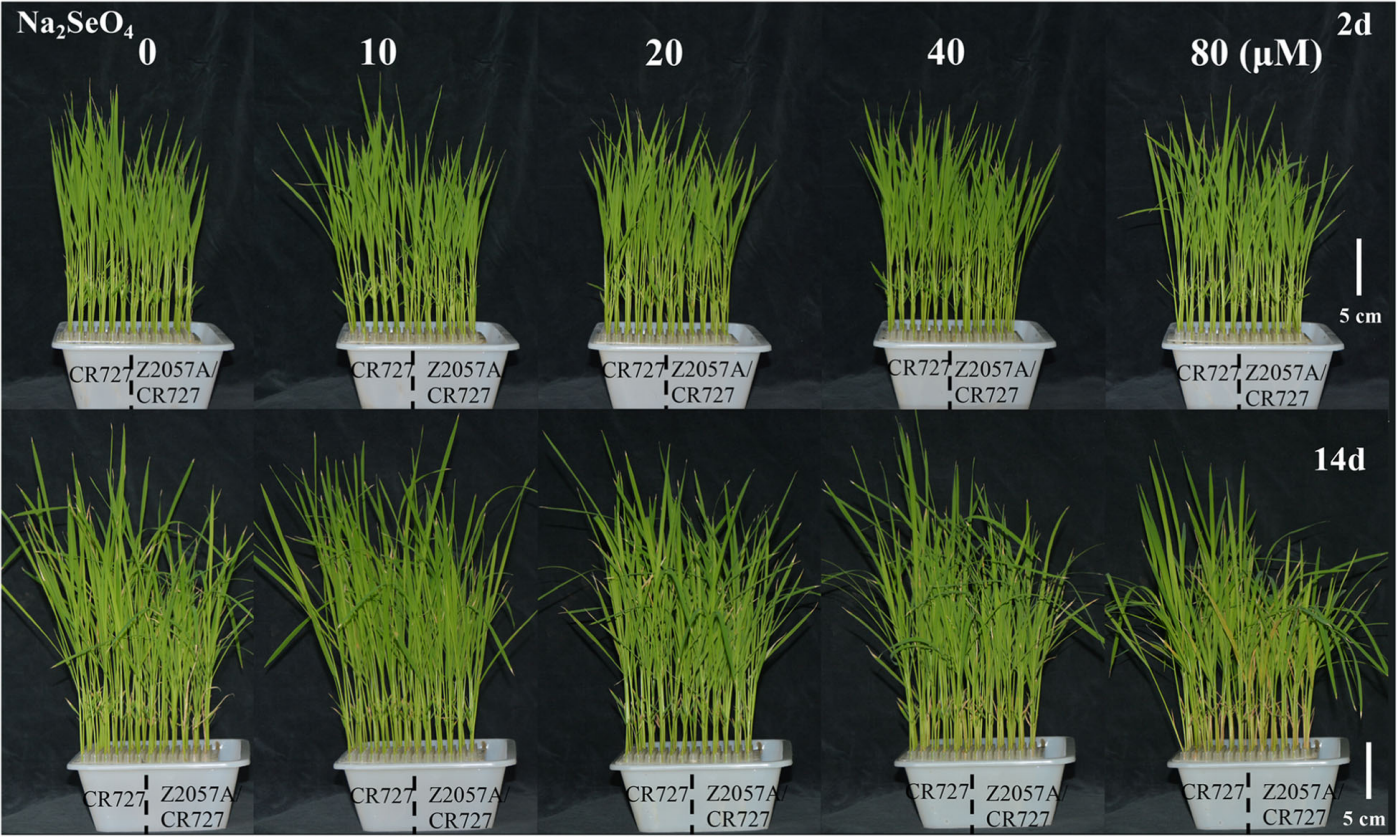
Phenotypes of aboveground architectures of grown seedlings of Se-rich rice and Se-free rice in presence of exogenous Se for 2 d (**a**) and 14 d (**b**). *Bar* = 5 cm.

To assess the effects of Se on rice morphology, we observed the alterations of the aboveground architectures in response to the application of a gradient of Se concentrations for 2 d and 14 d. Regardless of the treatment duration and the presence or absence of Se, the plant heights of the Se-free rice were always higher than the Se-rich rice (Fig. 2). Nevertheless, in the comparison to the Se-free rice, the height of Se-rich was impaired clearly in presence of a high concentration of Se (80 μM) at both 2 d and 14 d, indicating that the Se-rich exhibits a fast and primary response to exogenous Se toxicity (Fig. 2). There is no change in leaf color or appearance during short-term exposure to a series of Se concentrations (Fig. 2)a. On the contrary, although the plant height was still increasing, the long-term exposure under a high concentration of Se (80 μM) induced the yellowing of leave in both rice varieties which is in accordance with the decrease of chlorophyll (Fig. 1d and 2b) and inparticular, increasing senescence in leaves of the Se-rich rice were observed, demonstrating that the uptake of Se may result in cell death-related events.

### The production of reactive oxygen species in response to Se

**Fig. 3:**
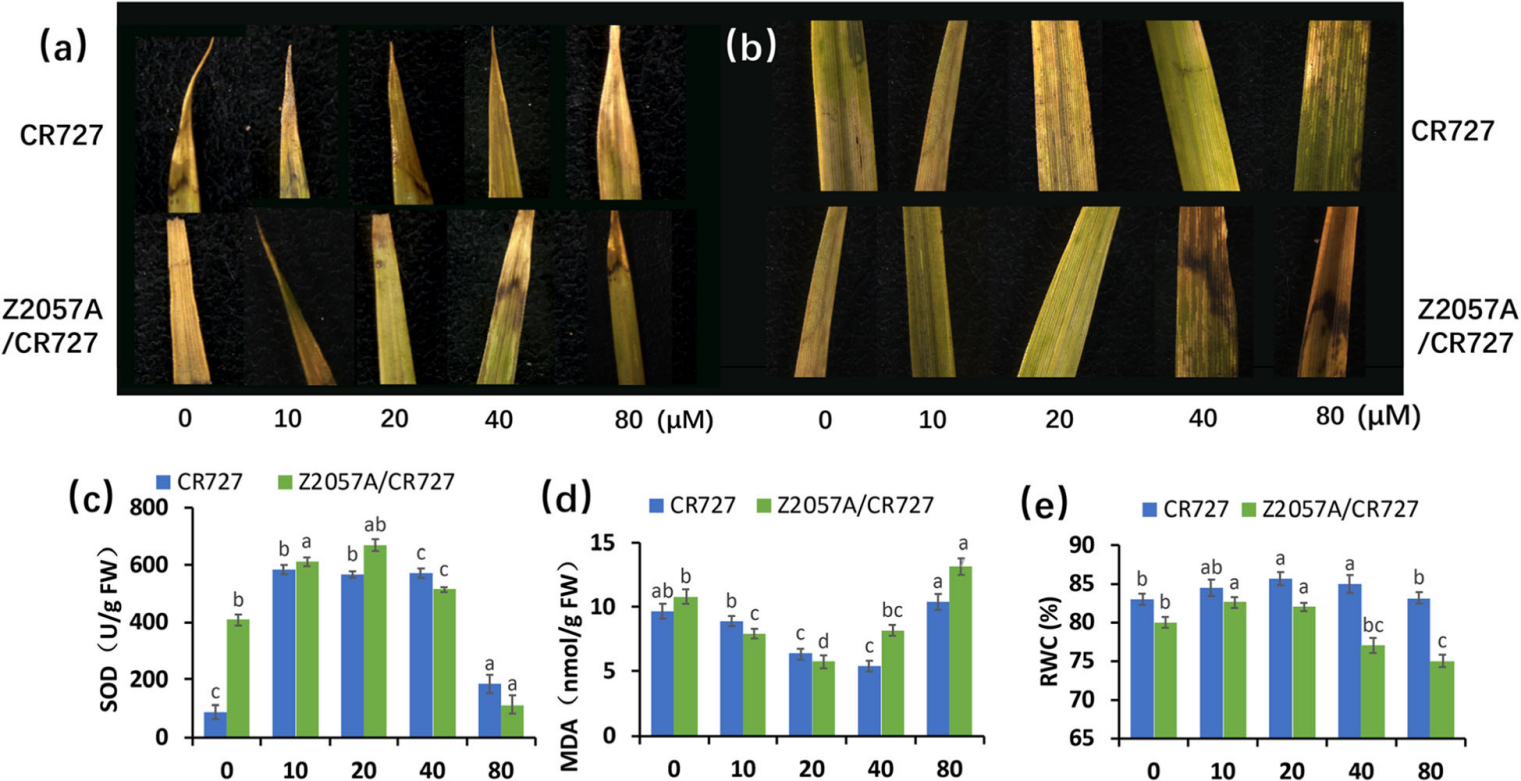
The determination of ROS in Se-rich rice and Se-free rice in presence of exogenous Se. (**a**) Nitrobluetetrazolium (NBT) staining of superoxide. (**b**) diaminobenzidine (DAB) staining of hydrogen peroxide (H_2_O_2_). (**c**) The activity of superoxide dismutase (SOD). (**d**) The content of methane dicarboxylic aldehyde (MDA). (**e**) The value of﻿ relative water content (RWC). Data presents mean ± SE (*n* = 3) and different letters indicate significant differences (*p* < 0.05)

ROS has been considered to mediate the primary response of plant resistance to environmental toxicity [22–24]. Thus, we reasoned that ROS signaling might be involved in rice response to Se toxicity during long-term exposure. To this end, we first investigated the production of the major ROS, superoxide (O^2−^) and hydrogen peroxide (H_2_O_2_) in response to Se by using histochemical staining methods, NBT staining and DAB staining, respectively. With the increasing of Se concentrations, the staining of NBT and DAB gradually spread to the entire leaf blade (Fig. 3a and b). Interestingly, the blast of accumulation of O^2−^ and H_2_O_2_ began from 40 μM Se in leaves of Se-rich rice, while it occurred earlier in those of Se-free rice.

To quantify the endogenous oxidative stress in accompany with the generation of ROS, the activity of SOD and the content of MDA in leaves were measured. Interestingly, the Se-rich rice had higher SOD activity than the Se-free rice in the absence of exogenous Se. The activity of SOD increased with the increasing of Se until a threshold (80 μM) where it plummeted reversely (Fig. 3c), which is matched to a dose-dependent manner presented at the whole-seedling level (Fig. 1 and 2). As the consequence, the content of MDA, an indicator of lipid peroxidation in plant cells [25, 26], declined in an opposite tendency to the increasing of the activity of SOD induced by Se in the exception of leaves at 80 μM Se where the activity of SOD was strongly inhibited, supporting that the elimination of ROS would be activated once its generation (Fig. 3d). Conclusively, the above evidence supported that ROS signaling is involved in the primary rice response to Se toxicity during long-term exposure.

Loss of water is one of the responses in leaves to external stresses in plants [22]. We next studied whether there are differences between Se-rich and Se-free rice, which may contribute to the diverse Se responses in these two varieties. The relative water contents (RWC) was determined. Intriguingly, while there are only slight changes of RWC in the Se-free rice, the values of RWC of the Se-rich rice in the presence of Se decreased remarkably at concentrations of 40 μM and 80 μM.

### The gene expression patterns of Se uptake- and transport-related factors in the application of exogenous Se

**Fig. 4:**
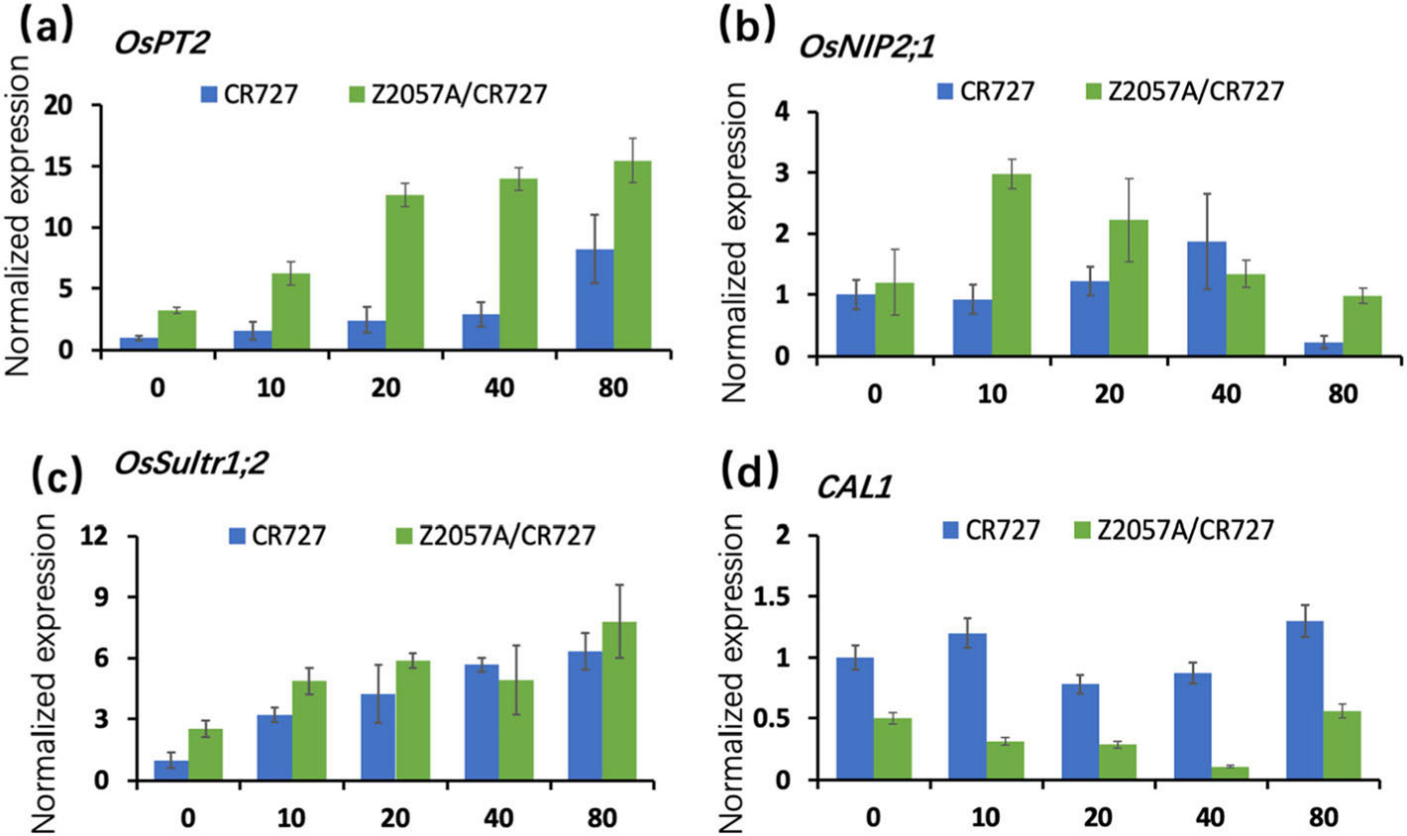
Expression patterns of ion transporter genes in roots of seedlings in the presence of exogenous Se. (**a**) the phosphate transporter gene *OsPT2*. (**b**) the Si influx transporter gene *OsNIP2;1*. (**c**) the sulfate transporter gene *OsSultr1;2.* (**d**) the Cd related transporter gene *CAL1*. The expression levels were normalized by the reference *g*enes *Actin1* and *EF1α*. Data presents mean ± SE (*n* = 6)

To understand the underlying mechanism of distinct Se-mediated physiological responses in Se-rich and Se-free rice varieties, we investigated the gene expression patterns of some key Se uptake- and transport-related factors in the application of exogenous Se by using real-time quantitative PCR (RT-qPCR). The expression of *OsPT2*, which encodes phosphate transports, was consistently up-regulated in the application of increasing concentrations of Se, indicating that OsPT2 might play a key role in the transportation of Se (Fig. 4a). Interestingly, the expression levels of *OsPT2* in the Se-rich rice were higher than in the Se-free rich in regardless of additions of Se (Fig. 4a). In absence of Se, there is no difference in the expression levels of the Si influx transporter encoding gene *OsNIP2;1* in the two rice varieties, by contrast, its expression pattern differed in the presence of Se (Fig. 4b). Compared to the control, there was no change of expression until a dramatically decrease in the highest concentration of Se in the Se-free rice (Fig. 4b). Speaking of its expression pattern in response to Se in the Se-rich rice, an increasing and reversely declining of expression were sequentially demonstrated in lower concentrations of Se (10 μM and 20 μM) and higher concentrations (40 μM and 80 μM), respectively, with a peak expression at point of 10 μM when the seedlings began to respond to Se-induced physiological growth (Figs. 1, 2, and 4b). In line with the previous assumptions [27, 28], the unique expression patterns presenting in the two rice varieties support that OsNIP2;1 may serve as a main positive regulator in Se transportation. Although the result showed that Se continuously induces the expression of *OsSultr1;2*, a sulfate transporter in presence of all concentrations of Se, we observed no obvious difference between the two rice varieties in those scenarios (Fig. 4c). Notably, the Se-rich rice accumulated enhanced expression level of *OsSultr1;2* than the Se-free rice in absence of Se (Fig. 4c), indicating that the activity of OsSultr1;2 may contribute to a relatively stunted shoot architecture of Se-rich rice (Figs. 1 and 2a) and positively mediate its sensitiveness to exogenous Se. Interestingly, the expression of *CAL1*,which encodes a Cd relative transporter maintains stable in absence and presence of Se in the Se-free rice, whereas it has been inhibited in the Se-rich rice (Fig. 4d). Conclusively, the gene expression data demonstrated that the uptake and transport of Se rely on known ion transporters, connecting to the assimilation processes of other elements.

## Discussion

**Fig. 5:**
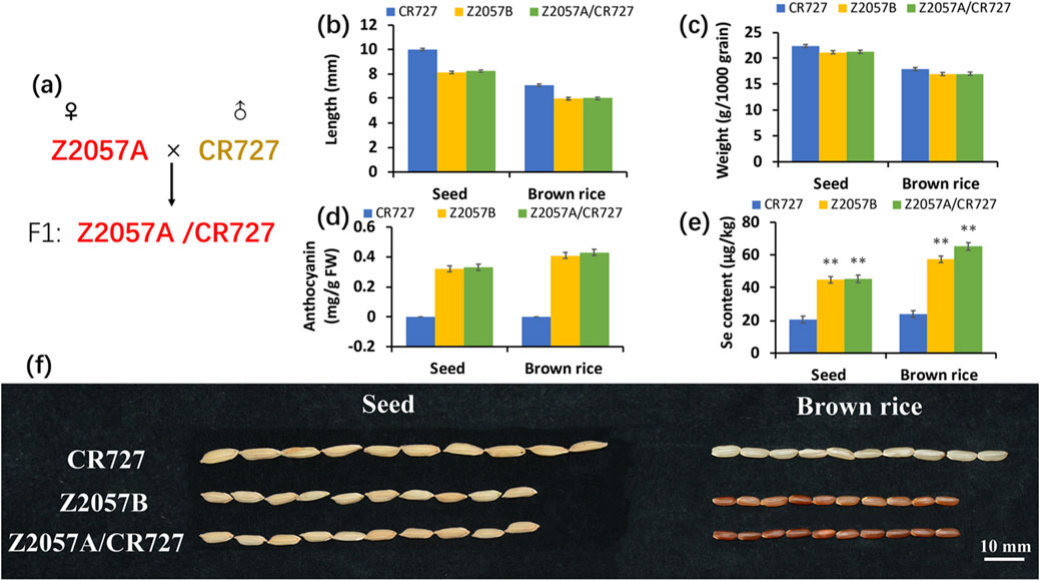
The characters of the Se-free rice CR727 and the Se-rich rice Z2057A/CR727. (**a**) The generating of Z2057A/CR727. (**b**) The length of the grain. (**c**) The weight of 1000 grains. (**d**, **e**) The contents of anthocyanin (**d**) and Se (**e**) in different rice. Data presents mean ± SE (*n* = 10, b; *n* = 3, **d** and **e**) and different letters indicate significant differences (*p* < 0.05)

Over 85% world widely of the rice is white-hulled, while the remaining is in a diversity of colored hulls, mainly in purple, black, and red [29]. In eastern Asia countries, China, Japan, South Korea and parts of Southeast Asia, colored rice has been consumed over centuries [30]. Colored rice is thought to possess abundant nutritional values. For instance, in vitro and in vivo studies have shown that extracts from colored rice have high antioxidant activity and free radical scavenging capability experiments [13, 31]. In this study, the red-colored Se-rich hybrid rice Z2057A/CR727 and one of its parental lines white-colored Se-free rice CR727 were included as experimental materials (Fig. 5a and f). The phenotypes and physiological effects of exogenous Se on the two rice varieties were studied. The results demonstrated that lower concentrations of Se supplement in a certain range are beneficial to the growth of rice, while the Se-rich hybrid rice is more sensitive to the applications of Se, suggesting that a dose-dependent regulatory mechanism is involved in rice responds to exogenous Se.

In consistence with the differences in grain colors, the content of anthocyanin is positively correlated to the content of Se in seeds and brown rice (Fig. 5b-f). Anthocyanin, which is one of the major products of flavonoid synthesis, is a class of water-soluble plant natural pigments [13, 32]. Anthocyanin has strong antioxidant activity to reduce and eliminate the effects of free radicals [33]. While the low concentration of Se promotes the growth of roots and leaves of rice, high Se inhibits [34]. SOD is a major antioxidant enzyme related to the scavenging of ROS, maintaining the balance of active oxygen metabolism to protect the entirety of membrane structure [35]; MDA is one of the final products of peroxidation of unsaturated fatty acids, the accumulation of which results indamages to cell membranes [36]. Nevertheless, the alterations of the SOD activity and the content of MDA present in a negative correlation; while the increasing concentrations of Se until 40 μM led to the enhancing of SOD activity and the declining of MDA accordingly. The SOD activity depressed dramatically at the highest concentration of Se (80 μM), as result, the content of MDA increased, supporting that rice release from exogenous Se toxicity by activating ROS signaling (Fig. 3a and b). Although it has been assumed that the exposure of high concentration of Se which is beyond a certain physiological threshold would be toxic to both rice varieties, the underlying mechanism of regulation of the generation of ROS and the activation of SOD arestill yet to be elucidated.

It has been reported that the loss-of-function mutant *sultr1;2* in *Arabidopsis* which retains other sulphate transporters, diminishes the capacity of taking up of selenate and exhibits elevated tolerance to Se in comparison to the wild-type plants [20, 21]. The presenting result (Fig. 4c) is in accordance with the previous study that the expression of *SULTR1;2* was promoted by the application of Se in *Arabidopsis* [12], providing evidence that SULTR1;2 acts as a conserved factor in mediating the assimilation of Se among plant species. In rice, the overexpression of *OsPT2* was able to increase the uptake of Se [37], while the capacity was greatly compromised in the *Ospt2* mutant [19]. The defect of the Si influx transporter gene *OsNIP2;1* resulted in a dismissal of distributions of Se in the shoots and xylem sap when the exogenous Se was supplied [18]. Our expression analysis showed that as low concentration as 10 μM of Se induced the expression of OsNIP2;1 in the Se-rich rice Z2057A/CR727, when there is no change in the control group of Se-free rice CR727, indicating that OsNIP2;1 may serve as a main positive regulator in Se transportation in rice plants and is associated with their endogenous Se level. On the other hand, previous studies have unraveled that the root exodermis and xylem parenchyma cells preferentially expressed Cd-related transporter gene CAL1 acts as a long-distance Cd transport in xylem vessels by chelating Cd in the cytosol,facilitating Cd secretion to extracellular spaces, hence balancing the concentrations of cytosolic Cd [38]. Whether there is a synergic or related effect in the assimilations of Cd and Se has been largely unknown. In this study, we investigated the expression patterns of *CAL1* in rice plants in the presence of Se. In contrast with the control Se-free rice which maintained a relatively stable expression of *CAL1*, its expression in the Se-rich rice decreased gradually to the lowest level in 40 μM of Se, indicating that CAL1 may negatively mediate the sensitiveness of Se application in the Se-rich rice. In addition, NRT1.1B, a member of the rice peptide transporter (PTR) family, is thought to improve the accumulation of Se in grains by facilitating ﻿selenomethinone (SeMet) translocation, provide novel insights into the breeding of Se-rich rice varieties [10]. In line with the previous report that roots of Se-hyper accumulator plants activate the expressions of ion transporters [39], the intervention on the expression activities of the abovementioned factors may contribute to the uptake capacity of Se for rice varieties.

On one hand, different rice varieties tend to exhibit different potentials in accumulating and accreting Se, and on the other hand, the exogenous Se stress oppositely determines the accumulation pattern of Se in different rice, therefore, it would be significant to understand how and what regulates the responses to the exposure of Se in rice varieties. The evidence showed that the organ-locally accumulation of Se in rice appeared in the following descending orders: leaves, stems, and roots in the presence of exogenous Se (Table 1). The determination data revealed that the increased accumulation of Se in seed and brown rice of the Se-rich rice Z2057A/CR727 is a trait that inherits from its parental line Z2057A (Fig. 5a and e).

**Fig. 6:**
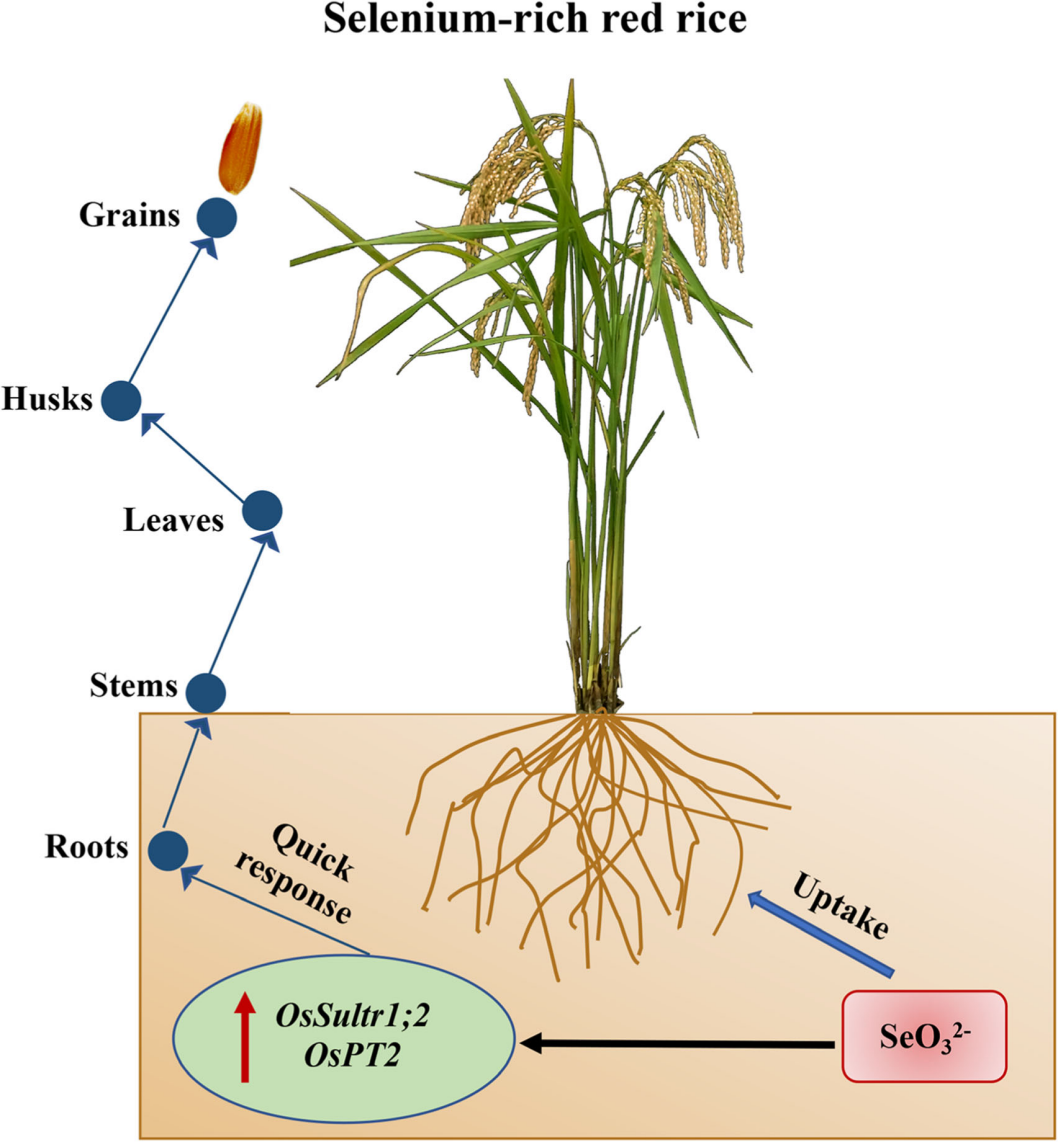
A working model of the uptake and assimilation of Se in the Se-rich rice. The uptake of soil selenate (SeO_3_^2−^) stimulates and relies on the expression of two transporter genes in roots, initiating a quick response to internalize Se gradually from the root, stem, leaf and husk to the grain, resulting in red-colored rice by increasing the content of anthocyanin

In current working model, the accumulation of Se was proposed to mainly relate to the translocation efficiency of this element (Fig. 6). In line with the previous study that the Se-rich rice adapts itself well only to low concentration of exogenous Se [40] (Fig. 1 and 2), our results supported that the Se-rich rice is more sensitive to the exposure of Se toxicity than the Se-free rice, rising those different management strategies are demanded for rice varieties based on case-by-case dissections. It would be promising to understand the underlying mechanism by identification and validation of involved genetic factors in the future.

## Conclusions

This study demonstrated that Se-rich rice is more sensitive to Se application. It can accumulate more Se from the root in low selenate environment while at high concentration of selenate application the effects were inverted. Besides, excessive selenate can cause phytotoxic effects on Se-rich rice plants by inducing chlorosis, dwarfness, reduced antioxidant contents, and exacerbating oxidative stress. In addition, we conclude that selenate enhanced gene ﻿transcription of phosphate transporter OsPT2 and sulfate transporters OsSultr1;2 to improve the uptake of Se. Meanwhile, hydroponically, the best Se concentration was 20 μM for Se-rich red rice root environment. ﻿Such findings can be useful to estimate the direct toxic effects of Se contamination on Se-rich rice in the field.﻿ To draw a sound conclusion, long-term field trials are still required, including risks and benefits analysis for various management strategies.

## Methods

### Plant materials, growth conditions and treatments healthy

The seeds of Se-free rice (*Oryza sativa* L.) CR727 and its hybrid progeny Se-rich red-grain rice Z2057A/CR727 were obtained from the collection of Demonstration Base for International Science & Technology Cooperation of Sichuan Province, Rice Research Institute, Sichuan Agricultural University, China with permission. Rice seeds were sterilized with 1% (v/v) NaClO for 20 min before rinsing 5 times in sterilized double-distilled water (ddH_2_O). To stimulate uniform germination, seeds were submerged in the sterilized ddH_2_O and incubated at 37 °C for 3 d in avoidance of light. Seedlings were hydroponically cultured in half-strength Kimura B nutrient solution (pH 5.5) at 25 °C, under the condition of 12 h light and 12 h dark [18]. The solution was renewed every 3 d to ensure fresh and nutrient stable during a long-term period [17]. In the exogenous Se stress test, the healthy seedlings were continuously growing in presence of a gradient of concentrations of sodium selenate (Na_2_SeO_4_, referring as Se hereafter) and mock control for additional 2 d or 14 d [41, 42]. Three independent biological replicates were included in phenotype observations and a variety of assays, while the fully expanded second leaves were harvested to determine growth-related parameters in the assessment of physiological and biochemical responses.

### Measurement water status of leaves

Relative water content (RWC) of leaves was introduced to quantify the water status of leaves. Fresh leaves were detached and weighed immediately to 0.1 g and designated as the fresh weight (FW). The same leaves were then soaked in ddH_2_O at 4 °C in darkness for 24 h before weight and designated as turgid weight (TW). After that, leaves were subjected to 80 °C for no less than 3d to get sufficient drying prior to weight and designated as for dry weight (DW). The RWC was calculated as (FW-DW)/(TW-DW) × 100% [43].

### Measurement of physiological parameters

Total leaf chlorophyll (Chl) content analysis, fresh leaves (0.1 g) were immersed in 10 ml of dimethyl sulphoxide (DMSO) in the dark for 48 h, and then the leaf extract was measured at 663 and 645 nm with a spectrophotometer as described by Arnon et al. [44, 45]. The contents of Chl (mg∙g^− 1^) were calculated by the following formula:

$$ Totalchlorophyll\left( mg\bullet {g}^{-1}\right)=\frac{\left(20.29{A}_{645}+8.05{A}_{663}\right)\times v\times 1000}{w} $$

The content of superoxide dismutase (SOD) and methane dicarboxylic aldehyde (MDA) were determined by a spectrophotometer-based method. SOD and MDA were first labelled by using a kit (A001–1 SOD and A003 MDA) from Nanjing Jiancheng Bioengineering Institute and according to the manufacturer’s instructions. After the reaction, the appearances of SOD and MDA were determined by subjecting the products to a UV-visible spectrophotometer equipped with cuvettes of 1 cm path length, respectively (T6S, Puxi, Co., Ltd., Beijing, P. R. China). After harvesting the values, the activity of SOD and content of MDA were calculated according to the following formula

$$ Total\  SOD\  Activity\ \left(U\bullet {g}^{-1}\right)=\left(\frac{OD_A-{OD}_B}{OD_A}\right)\div 50\%\times \frac{V_{Reaction\ Total}}{V_{Sample\ Fluid}}\div \frac{W_{tissue/g}}{V_{homogenate/ ml}} $$

$$ Tissue\  MDA\  Content\left( nmol\bullet {g}^{-1}\right)=\left(\frac{OD_{Sample}-{OD}_{Sample\ Blank}}{OD_{Standard}-{OD}_{Standard\ Blank}}\right)\times 10\left( nmol\bullet {ml}^{-1}\ \right)\div {W}_{tissue/g} $$

### Histochemical analysis of reactive oxygen species

To detect the presence of superoxide in leaves, the leaves were incubated in the staining solution of nitrobluetetrazolium (NBT, 0.1%) as described previously [46]. To detect the accumulation of hydrogen peroxide (H_2_O_2_), Rice leavesof seedlings after 14 d exogenous treatment with or without (control) Se were collected and stained in a 3,3′-diaminobenzidine-HCl (DAB, 1.0%) solution as described previously [46].

### Measurements of se and anthocyanin content

An atomic fluorescence spectrophotometer was applied to determine the Se content as described previously [47, 48]. Briefly, after grinding into fine powder, 0.5 g samples were weighted and filled into a glass vial containing a pre-prepared solution of 9 ml HNO_3_ and 1 mL HClO_4_. The sampling solutions were ultrasonicated with a fixed setup of parameters (temperature, duration, and frequency) at 20 °C, 4 h, and 100 Hz, respectively, following by a digestion process in the presence of HNO_3_ in a 180 °C-electric hot plate (EH20A Plus, Labtech, USA). The digested products were then diluted with a suitable amount of 37% hydrochloric acid to reduce Se (VI) into Se (IV) within a consistent temperature of 90 °C, as result, a whitish concentrated solution was generated in a volume of 1 ml due to the heating-induced evaporation. The measurements were carried out in an atomic fluorescence spectrophotometer (RGF-6800, Bohui Co., Ltd., Beijing, China). The values were put into the following formula to calculate the content of Se (mg/kg):

$$ Se\  content=\frac{\left(C-{C}_0\right)\times V\times 1000}{m\times 1000\times 1000}, $$

where *C* is the measured value of Se concentration in the digested solution (ng/mL); *C*_*0*_ is the measured value of Se concentration in the control group (without any samples, ng/mL); *m* is the mass of sample; *V* is the total volume of digested solution. The measurements were performed in triplicate.

The extraction of anthocyanin from rice was determined as described previously [13]. The content of anthocyanin was calculated by putting the values into the following formula:

$$ Total\ anthocyanins\ \left( mg\bullet {g}^{-1}\right)=\frac{\left[\left({A}_{530}-{A}_{620}\right)-0.1\left({A}_{650}-{A}_{620}\right)\right]\times V}{\varepsilon \times m\times 1000}\times M $$

where *V* is the total volume of extraction solution (mL); *ε* is the anthocyanin molar extinction coefficient (4.62 × 10^6^); *m* is the mass of sample (g); *M* is the molecular weight of anthocyanin. The measurements were performed in triplicate. The measurements were performed in triplicate.

### Relative genes expressionanalysis

According to the qRT-PCR method [3], mRNA of two-week treatment rice seedling roots was extracted by using the TRI pure reagent kit (Aid Lab). Primers used in these assays synthesized by Qingke Company (QingkeZixi Co., Ltd., Chengdu, China) are listed in Table 2, and the expression levels were normalized to those of the *Actin1* and *EF1α* as indicated. qRT–PCR was carried out on a CFX96 Real-Time PCR Detection System (Bio-Rad) with SYBR Green Master Mix (2X) to monitor double-stranded DNA synthesis using a three-step PCR cycling program (95 °C for 15 s, followed by 40 cycles of 95 °C for 15 s, 55 °C for 15 s and 72 °C for 20 s). The 2^−ΔΔCT^ method was used to calculate the expression levels of target genes [49, 50].

**Table 2:** Oligo sequences used in this study

Gene	Primer sequences
*Actin1* (Os03g0718100)	FW: TCCATCTTGGCATCTCTCAG RV: GTACCCGCATCAGGCATCTG
*EF1α*(Os03g08010)	FW: TTTCACTCTTGGTGTGAAGCAGAT RV: GACTTCCTTCACGATTTCATCGTAA
*OsPT2*(Os03g05640)	FW: AAACTTCCTCGGTATGCTCATG RV: ATGTTTATGACATCACGCTTGG
*OsNIP2;1*(Os02g51110)	FW: AACATCCAAGTGTGATAGGACG RV: ACACAAAGACGTAGCTAGTGAT
*OsSultr1;2*(Os03g0195500)	FW: TCAAAGAAGAACCCGCTAGATT RV: GCAATTCCAAGGAAGCCTTTAA
*CAL1*(Os02g0629800)	FW: AGTCGCGTGTTCTCCTTTGT RV: AGTCGCGTGTTCTCCTTTGT

### Statistical analysis

All the phenotype observations and physiological assays were performed in biological triplicates. Data were presented as mean ± standard error (SEM). Statistical analysis was performed with an SPSS 24.0 statistical package (SPSS Inc., Chicago, IL, USA). One-way ANOVA was carried out with multiple comparisons using Duncan’s test to evaluate significant differences at 0.05 probability level.

## Data Availability

The datasets used and analyzed during the current study available from the corresponding author on reasonable request.

## Abbreviations

Cd: cadmium
Chl: Chlorophyll
DAB: 3,3′-Diaminobenzidine
ddH_2_O: Double distilled water
H_2_O_2_: Hydrogen peroxide
MDA: Methane dicarboxylic aldehyde
NBT: Nitrobluetetrazolium
Pi: Phosphate
qRT-PCR: Quantitative reverse transcription polymerase chainreaction
ROS: Reactive oxygen species
Se: Selenium
Si: Silicon
SOD: Superoxide dismutase
